# Production of Mono-Hydroxylated Derivatives of Terpinen-4-ol by Bacterial CYP102A1 Enzymes

**DOI:** 10.4014/jmb.2310.10018

**Published:** 2023-12-01

**Authors:** Jeong-Hoon Kim, Chan Mi Park, Hae Chan Jeong, Gyeong Han Jeong, Gun Su Cha, Sungbeom Lee, Chul-Ho Yun

**Affiliations:** 1 School of Biological Sciences and Biotechnology, Graduate School, Chonnam National University, Gwangju 61186, Republic of Korea; 2 School of Biological Sciences and Technology, Chonnam National University, Gwangju 61186, Republic of Korea; 3 Research Division for Biotechnology, Advanced Radiation Technology Institute (ARTI), Korea Atomic Energy Research Institute (KAERI), Jeongeup 56212, Republic of Korea; 4 Namhae Garlic Research Institute, Namhae 52430, Republic of Korea; 5 Department of Radiation Science and Technology, University of Science and Technology, Daejeon 34113, Republic of Korea; 6 Institute of Synthetic Biology for Carbon Neutralization, Chonnam National University, Gwangju 61186, Republic of Korea

**Keywords:** Cytochrome P450, CYP102A1 mutant, carbon hydroxylation, cyclic monoterpene, terpinen-4-ol

## Abstract

CYP102A1 from *Bacillus megaterium* is an important enzyme in biotechnology, because engineered CYP102A1 enzymes can react with diverse substrates and produce human cytochrome P450-like metabolites. Therefore, CYP102A1 can be applied to drug metabolite production. Terpinen-4-ol is a cyclic monoterpene and the primary component of essential tea tree oil. Terpinen-4-ol was known for therapeutic effects, including antibacterial, antifungal, antiviral, and anti-inflammatory. Because terpenes are natural compounds, examining novel terpenes and investigating the therapeutic effects of terpenes represent responses to social demands for eco-friendly compounds. In this study, we investigated the catalytic activity of engineered CYP102A1 on terpinen-4-ol. Among CYP102A1 mutants tested here, the R47L/F81I/F87V/E143G/L188Q/N213S/E267V mutant showed the highest activity to terpinen-4-ol. Two major metabolites of terpinen-4-ol were generated by engineered CYP102A1. Characterization of major metabolites was confirmed by liquid chromatography-mass spectrometry (LC-MS), gas chromatography-MS, and nuclear magnetic resonance spectroscopy (NMR). Based on the LC-MS results, the difference in mass-to-charge ratio of an ion (m/z) between terpinen-4-ol and its major metabolites was 16. One major metabolite was defined as 1,4-dihydroxy-*p*-menth-2-ene by NMR. Given these results, we speculate that another major metabolite is also a mono-hydroxylated product. Taken together, we suggest that CYP102A1 can be applied to make novel terpene derivatives.

## Introduction

Terpinen-4-ol is a cyclic monoterpene and the primary component of essential tea tree oil (*Melaleuca alternifolia*). It makes up 30–40% of tea tree oils [1, 2]. Terpinen-4-ol has several important therapeutic properties, including antiviral [3, 4], anti-inflammatory [5], and analgesic properties [6]. Terpinen-4-ol has antibacterial effects against *Pseudomonas aeruginosa*, *Escherichia coli*, *Staphylococcus aureus*, and *Streptococcus agalactiae* [7, 8]. It also has antifungal effects against *Candida* spp. [9], dermatophytes and filamentous fungi [10], and *Aspergillus ochraceus* [11]. This monoterpene is colorless to slightly yellow and a fragrance ingredient (pepper/woody and earthy/musty) [12, 13]. Its properties as a fragrance ingredient are suitable for products utilizing fragrance, and terpinen-4-ol has been used in cosmetics such as perfumes, shampoos, soaps, and cosmetics [14]. Terpinen-4-ol has the potential for utilization as a pesticide, as it shows an insecticidal effect against flour beetles such as *Tribolium confusum* [15]. Because the demand for eco-friendly insecticides has increased to overcome various negative effects from synthetic insecticides, such as environmental destruction, researchers are attempting to replace synthetic pesticides, which trigger environmental pollution, with naturally derived compounds [16, 17].

The representative reaction of cytochrome P450 (CYP or P450) is the monooxygenation that transfers an oxygen atom from molecular oxygen to a variety of biological substrates, reducing the second oxygen atom by two electrons to a water molecule [18-21]. CYP is necessary for primary defense against xenobiotics, metabolism of drugs, and the production of cholesterol, steroids, prostacyclins, and thromboxane A2 [22, 23]. CYP102A1 from *Bacillus megaterium* is a natural fusion protein that consists of a heme-containing monooxygenase domain and reductase domain. Thus, CYP102A1 requires only NADPH and oxygen to react with substrates [24]. CYP102A1 produces the same metabolites of human drugs catalyzed by human-CYP [25, 26]. CYP102A1 can be expressed in recombinant *E. coli*. Additionally, CYP102A1 is able to produce various regioselective and stereoselective compounds [27-31]. These properties of CYP102A1 have attracted attention in various industrial areas. Regio-and enantioselective hydroxylation of terpenoids are difficult reactions to achieve through chemical synthesis because of the need for expensive catalysts, side reactions, and low yields [32]. However, CYP enzymes are utilized in bioconversion processes for regio- and stereo-selective oxygen introduction under mild reaction conditions [33]. In particular, soluble bacterial CYP enzymes are advantageous for production. Bacterial CYPs have been characterized by high catalytic turnover rates, high expression level in heterologous hosts, low costs of production, and ease of engineering. Diverse enzyme-engineering methods for improving catalytic activity and the results of terpenoid conversions using microbial CYP have been suggested [32].

CYP102A1 can react with terpenoids such as limonene [34-36], ionones [37-40], and steroids [41-43]. Modifying terpenoids’ base structure can trigger biological properties [44]. For example, hydroxylating terpenoids affects properties such as efficacy, toxicity, volatility, and solubility. CYPs can produce various derivatives of limonene [44]. In particular, various oxidized compounds were detected from reaction products of limonene derivatives catalyzed by engineered CYP102A1 [35, 36, 45, 46], and terpinen-4-ol has been produced from limonene by *Aspergillus niger* [47, 48]. Chemical synthesis of terpinen-4-ol from limonene has also been reported [49, 50]. Interestingly, human CYP1A2, CYP2A6, and CYP3A4 catalyze the oxidation of (+)-terpinen-4-ol. CYP2A6 and CYP3A4 were determined to be major enzymes involved in the oxidation of (+)-terpinen-4-ol by human liver microsomes to produce (+)-(1R,2S,4S)-1,2-epoxy-*p*-menthan-4-ol, (+)-(1S,2R,4S)-1,2-epoxy-*p*-menthan-4-ol, and (4S)-*p*-menth-1-en-4,8-diol [51]. On the other hand, CYP2A6 was the major CYP for oxidation of (-)-terpinen-4-ol to produce (−) (1S,2R,4R)-1,2-epoxy-*p*-menthan-4-ol, although CYP1A2, CYP2A6, and CYP3A4 also showed some activity concerning the oxidation of (-)-terpinen-4-ol [52]. However, as far as we know, there are no reports regarding the oxidation of terpinen-4-ol by CYP102A1.

It should be noted that terpinen-4-ol and its derivatives can be developed as drug candidates for cancer treatment. Terpinen-4-ol shows significant therapeutic effects on gastrointestinal [53], lung [54], and colorectal [55] cancers. Therefore, hydroxylated derivatives of terpinen-4-ol such as 1,4-dihydroxy-*p*-menth-2-ene, which is developed in this work using CYP as a catalyst, have important potentials for anticancer drug candidates. In addition, terpinen-4-ol is available in both enantiomeric (−)-*R*- and (+)-*S*- forms. Therefore, it and its hydroxylated derivatives can be used as building blocks for the organic synthesis of related high value-added compounds. Also, they can be used a chiral auxiliary in asymmetric syntheses like the case of (+)-*R*-limonene [56].

**Fig. 1:**
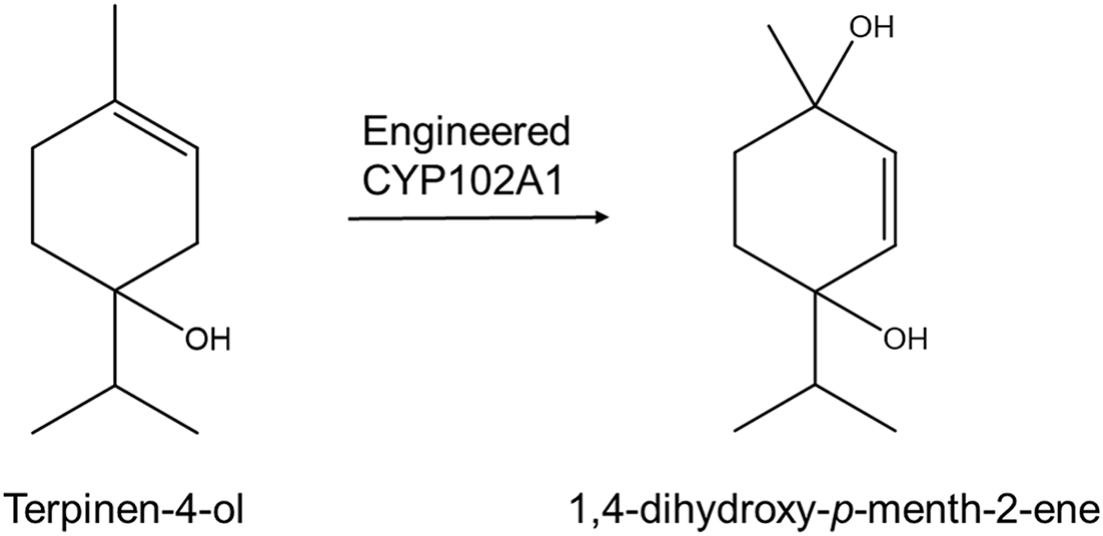
Scheme for 1,4-dihydroxy-*p*-menth-2-ene production by CYP102A1 mutants.

The objective of this study was to make novel terpene derivatives using engineered CYP102A1 and to suggest an efficient enzymatic strategy for generating terpene derivatives. We found that engineered CYP102A1 produced 1,4-dihydroxy-*p*-menth-2-ene from terpinen-4-ol (Fig. 1). We expect that engineered CYP102A1 can be a good catalyst to generate novel terpene derivatives.

## Materials and Methods

### Materials

Terpinen-4-ol (racemic), glucose-6-phosphate, glucose-6-phosphate dehydrogenase, and β-nicotinamide adenine dinucleotide phosphate (NADP^+^) were purchased from Sigma-Aldrich. High-performance liquid chromatography (HPLC) -grade acetonitrile was purchased from Fisher Scientific. HPLC-grade formic acid was purchased from Duksan Pure Chemicals. All of the other chemicals used were of the highest grade commercially available.

Originally, chromosomal DNA of *B. megaterium* KCCM 11745 for cloning of CYP102A1 wild type was obtained from Korean Culture Center of Microorganisms (KCCM, Republic of Korea). The gene of CYP102A1 wild-type (WT) was cloned and expressed in *E. coli* as described previously [57].

### Screening of CYP102A1 Mutant Activity toward Terpinene-4-ol

Mutants of the CYP102A1 gene were obtained from earlier work [57-60]. In this study, we selected M179 (R47L/F81I/F87V/E143G/L188Q/N213S/E267V) for bioconversion of 1,4-dihydroxy-*p*-menth-2-ene from terpinene-4-ol. The reaction mixture (V_t_ = 250 μl) contained 0.2 μM of CYP102A1 mutants in 100 mM potassium phosphate buffer (pH 7.4) and 0.5 mM of terpinen-4-ol. The stock of terpinen-4-ol was made in dimethyl sulfoxide (DMSO) up to 250 mM. The final concentration of DMSO in enzyme reaction was 2% (v/v). The NADPH generation system (NGS) was composed of 10 mM glucose-6-phosphate, 0.5 mM NADP^+^, and 1.0 IU yeast glucose-6-phosphate dehydrogenase per ml (final concentrations). After the reaction mixture was pre-incubated for 5 min at 37°C, NGS was added to initiate the reaction. The mixture was incubated for 10 min before the reaction was stopped by adding 25 μl of HCl (2 M). After mixing vigorously, the reaction mixture was centrifuged at 3500 ×*g* for 10 min. The mixture was purified using a 0.2 μm polyvinylidene fluoride (PVDF) filter purchased from Pall (USA) and transferred to vials. The filtrate was analyzed by HPLC (Shimadzu, Japan) equipped with liquid chromatograph (LC-20AD), auto sampler (SIL-20A), and UV/VIS detector (SPD-20A). All devices were purchased from Shimadzu. The mobile phase consisted of A (0.1% of formic acid in water) and B (0.1% of formic acid in acetonitrile), which was programmed as follows: For the isocratic B (40%), the flow rate was 0.5 ml/min. The detection wavelength was set as 200 nm, and the injection volume was 30 μl. Separation was achieved using an Agilent C18 column (5 μm, 4.6 × 150 mm). The metabolite quantitation was done by comparing the peak areas of metabolites and substrates.

### Optimization of Enzyme Reaction to Terpienen-4-ol

Various pH conditions were tested to find an optimal condition. The reaction mixture contained 0.2 μl of mutant M179 in a range of potassium phosphate buffer (pH 5–9) and terpinen-4-ol. The reaction was initiated by adding NGS, and the reaction mixture was incubated for 10 min before the reaction was stopped by 25 μl of HCl (2 M). The mixture was centrifuged for 10 min at 3,500 ×*g* and purified using a 0.2 μm PVDF filter.

Various temperature conditions were tested. After adjusting the pH to 7, the reaction mixture was prepared in the same way as described above. The reaction was carried out for 30 min at 25–45°C before it was stopped by 25 μl of HCl (2 M). The mixture was centrifuged for 10 min at 3,500 ×*g* and purified using a 0.2 μm PVDF filter. HPLC was used to analyze pH and temperature effects on metabolite formation.

### Identification of Terpinen-4-ol Metabolites by Liquid Chromatography-Mass Spectrometry Analysis

Liquid chromatography-mass spectrometry (LC-MS) was used to identify the products of terpinen-4-ol formed by CYP102A1 mutants. Preparation of samples for analysis was as follows: After the reaction mixture and NGS were incubated for 5 min, NGS was added to initiate the reaction; 600 μl of cold ethyl acetate was added to the mixture and vigorously mixed for 30 sec. The mixture was centrifuged at 3,500 ×*g* for 20 min, and 300 μl of supernatants were collected. The 300 μl sample was evaporated using N_2_ gas and dissolved with a mobile phase.

LC-MS analysis was performed on a Thermo Scientific Accela and TSQ Quantum Access MAX system with the heated electrospray ionization interface and a HESI II probe (Thermo Fisher Scientific, USA). Thermo Xcalibur Acquisition and Processing Version 2.2 was employed. An aliquot (5 μl) of sample was separated using ZorBax SB-C18 (4.6 × 250 mm, 5 μm, 80 Å; Agilent Technologies). The heated capillary and vaporizer temperatures of the mass spectrometer were both tuned to 320°C, whereas the nitrogen sheath gas and auxiliary gas pressures were each optimized at 40 and 15 psi. The spray voltage and scan speed were 3700 V and 0.8 spectra per second, respectively. The sample was analyzed using positive mode.

### Identification of Terpinen-4-ol Metabolites by Gas-Chromatography-Mass Spectrometry Analysis

Samples for gas chromatography (GC) analysis were constructed as follows: The reaction mixture (Vt = 250 μl) contained 0.4 μM of M179 in 100 mM potassium phosphate buffer (pH 7.4) and 0.5 mM of terpinen-4-ol. NGS was added into reaction mixture to initiate the reaction. The mixture was incubated for 1 h and extracted by adding 600 μl of cold ethyl acetate. The mixture was mixed vigorously and centrifuged at 3,500 ×*g* for 10 min. Then, 150 μl of ethyl acetate was collected and analyzed by GC as follows:

GC-MS analysis of metabolites of terpinen-4-ol was performed using Shimadzu GC system equipped with GCMS-QP2020 NX (GC-MS), GC-2030 (GC), and AOC-30i (auto injector). The sample (1 μl) was injected into the DB-5MS UI column (30 m × 0.25 mm, 0.25 μm Agilent). The injector temperature was adjusted to 250°C, and the initial oven temperature was kept at 50°C for 5 min, then raised to 250°C at a rate of 10°C /min and held for 10 min. The mass spectra were analyzed with a scanning range from 40 to 400 m/z.

### Identification of Terpinen-4-ol Metabolites by Nuclear Magnetic Resonance Spectroscopy

NMR analysis was conducted to identify the metabolites of terpinen-4-ol. M1 and M2 were separated by HPLC, collected, and frozen (–80°C) overnight. The samples were evaporated by freeze-dryer (Operon, Republic of Korea).

^1^H and ^13^C nuclear magnetic resonance (NMR) spectra were measured on an Avance NEO-600 instrument (Bruker, Germany) operated at 600 and 150 MHz, respectively. Chemical shifts are given in δ (ppm) values relative to those of the solvent CDCl_3_ (δ_H_ 7.26; δ_C_ 77.1) on a tetramethylsilane scale. Standard pulse sequences programmed into the instruments were used for each two-dimensional measurement. The *J*_CH_ value was set at 8 Hz in heteronuclear multiple bond connectivity (HMBC) spectra.

### Kinetic Parameters of Terpinen-4-ol Hydroxylation

Various concentrations of terpinen-4-ol (0.5–10 mM) and 0.2 μM CYP102A1 in 100 mM potassium phosphate buffer (pH 7.4) were used to determine the kinetic parameters of CYP102A1 mutants. After the mixture and NGS were pre-incubated for 5 min, NGS was added in mixture to initiate reaction for 10 min at 30°C. The reaction was stopped by adding 25 μl of HCl (2 M), and the mixture was centrifuged for 10 min at 3,500 ×*g* and purified by 0.2 μm PVDF filter. The filtrate was analyzed by HPLC. GraphPad Prism software (GraphPad Software, USA) was used to determine the kinetic parameters.

### Spectral Binding Titration

Spectral binding titration of terpinen-4-ol to the CYP102A1 enzymes was conducted with a Shimadzu 160PC spectrometer. To determine M179 binding affinity, various concentrations of terpinen-4-ol were added to 2 μM of M179 in a 100 mM phosphate buffer at pH 7.4. A shift of absorbance spectrum with every addition of substrate was observed between 350 and 500 nm. The dissociation constant from spectral results was determined using GraphPad Prism Software.

### Time Course-Dependent Formation of Terpinen-4-ol Metabolites

A time course analysis was conducted to investigate yield and productivity. Two concentrations of substrates (5 mM and 10 mM of terpinen-4-ol) were used for the experiments. The reaction mixture contained 0.2 μm of M179 in 100 mM potassium phosphate buffer (pH 7) and terpinen-4-ol. The reaction was initiated by adding NGS (final concentrations: 10 mM glucose-6-phosphate, 0.5 mM NADP^+^, and 1.0 IU yeast glucose-6-phosphate dehydrogenase per ml), and incubated from 30 min to 6 h. After centrifuging for 10 min at 3,500 ×*g*, the mixture was purified by a 0.2 μm PVDF filter. The filtrate was analyzed by HPLC, and metabolite quantitation was done by comparing the peak areas of metabolites and substrates.

## Results and Discussion

### Screening of CYP102A1 Mutant Activity toward Terpinen-4-ol

**Fig. 2:**
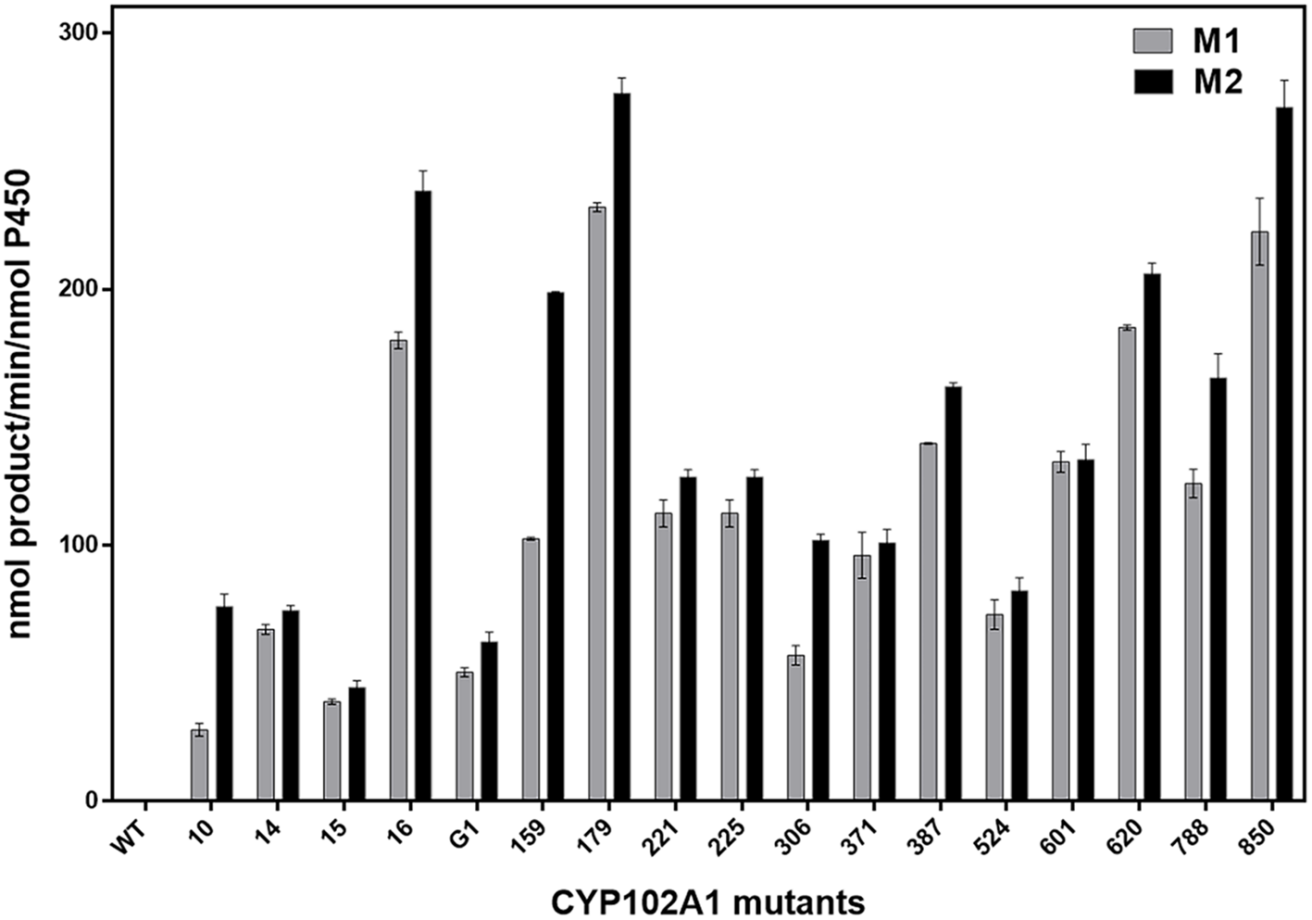
Screening of CYP102A1 mutants with terpinen-4-ol. The mixture contained 0.2 μM of CYP102A1 mutants and 5 mM of terpinen-4-ol in 100 mM potassium phosphate buffer (pH 7.4). The mixture with NGS was incubated for 10 min at 30°C.

**Fig. 3:**
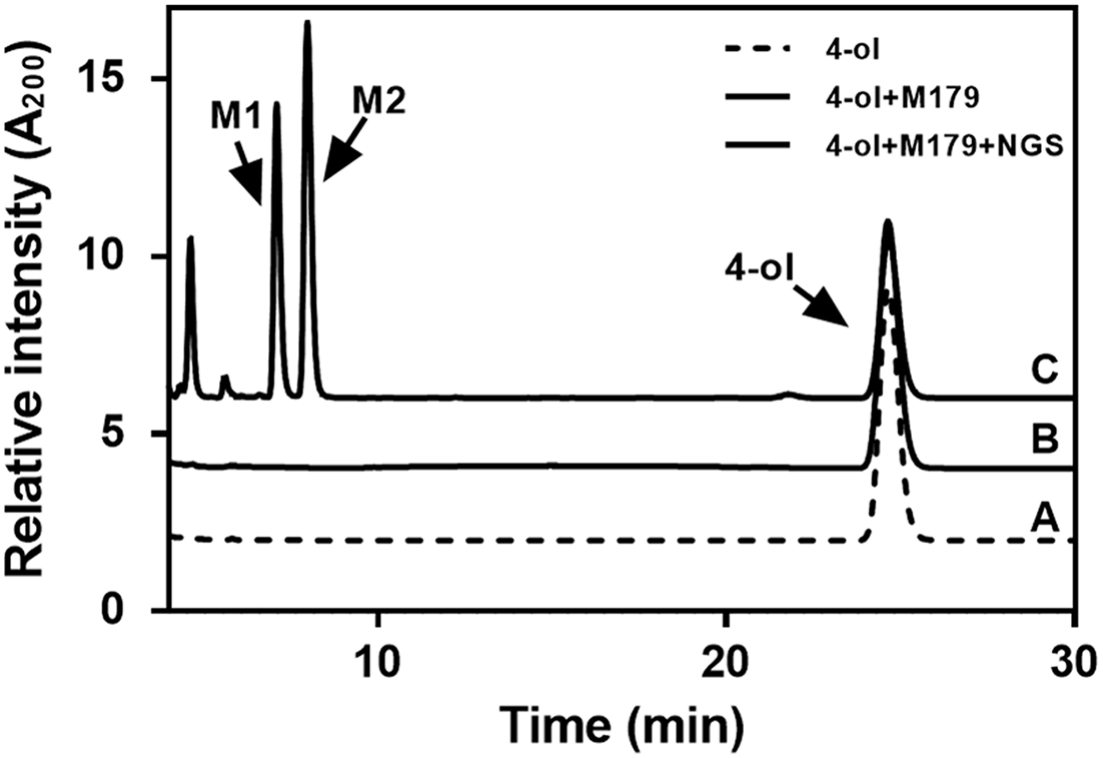
HPLC chromatogram of terpinen-4-ol and its major products generated by CYP102A1 mutant M179. The mixture contained 0.2 μM of CYP102A1 M179 and 5 mM of terpinen-4-ol in 100 mM potassium phosphate buffer (pH 7.4). The mixture with NGS was incubated for 1 h at 30°C.

The oxidation activity of CYP102A1 mutants concerning terpinen-4-ol was screened using WT CYP102A1 and its 25 mutants (Fig. 2, Table S1). The mutants used in this study were obtained from previous studies [57-60]. The HPLC chromatogram of metabolites (M1 and M2) and terpinen-4-ol were eluted at retention times 7.2 min, 8 min, and 24.7 min, respectively. Only when terpinen-4-ol, CYP102A1, and NGS were combined, two major metabolites (M1 and M2) were produced (Fig. 3).

Even though the WT CYP102A1 did not show apparent catalytic activity toward terpinen-4-ol (less than 0.1 min^–1^), some mutants have catalytic activity with terpinen-4-ol. Except for eight mutants that had no catalytic activity with terpinen-4-ol, mutants having apparent catalytic activity produced M2 more than M1. Mutants M179 and M850 produced two products with more than 200 min^–1^ (Fig. 2), and CYP102A1 M179 was selected for further study.

### Optimal Conditions for Enzyme Reaction

Various pH and temperature conditions were investigated to optimize the conditions for enzyme reaction. The formation of metabolites according to pH change is shown in Fig. S1. We found that pH 7 showed the highest activity among tested pH values, and pH 7 was used for the activity assay of CYP102A1 mutants.

The effect of temperature on the formation of metabolites of terpinen-4-ol was also investigated. In the range of 25–45°C (Fig. S2). We found that 25–30°C showed the highest activity, and enzyme activity decreased sharply from 40°C. Therefore, 30°C was used for enzymatic reactions.

### Characterization of Metabolites of Terpinen-4-ol by LC-MS

**Fig. 4:**
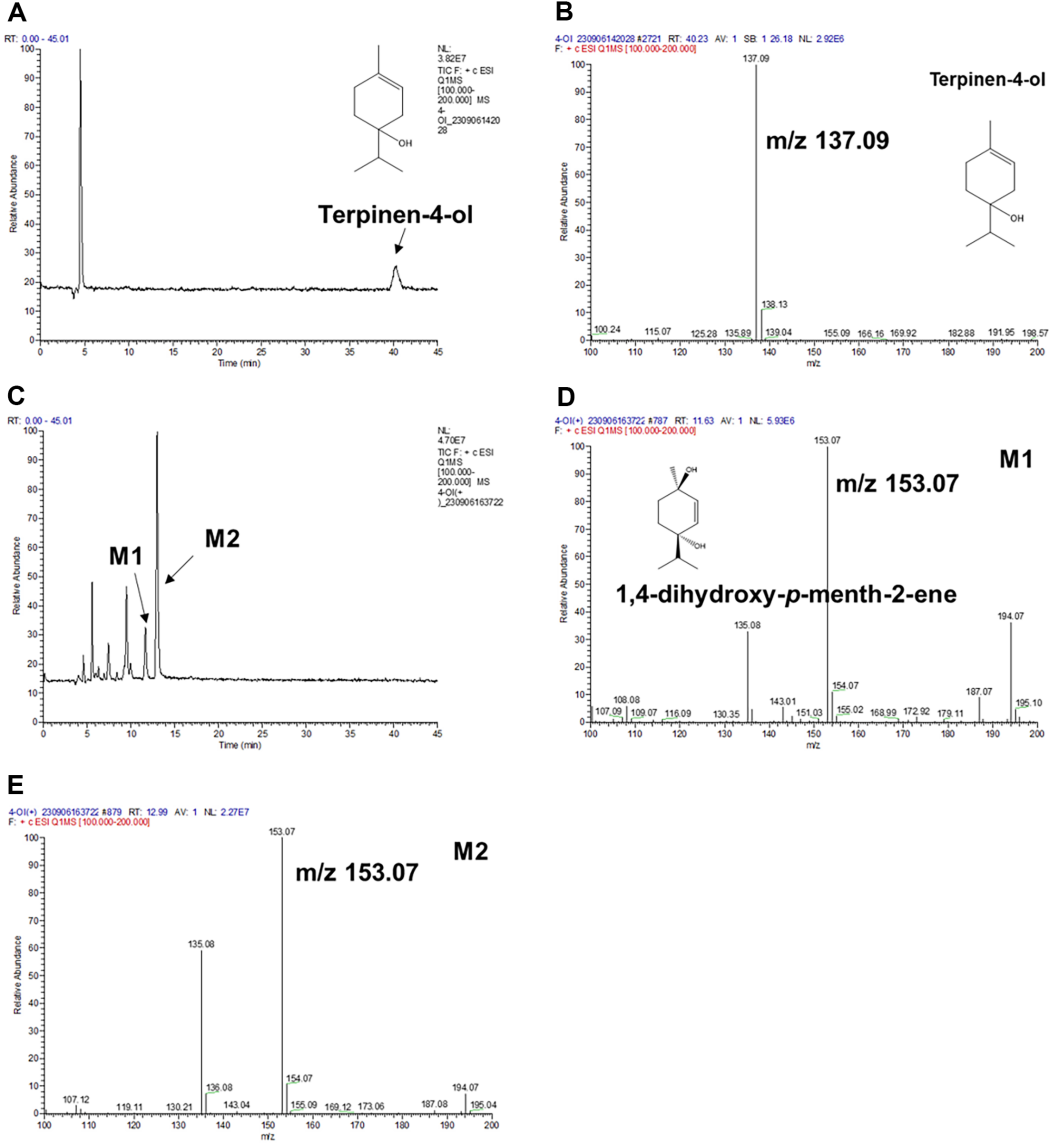
LC-MS/MS analysis of terpinen-4-ol and its metabolites produce by CYP102A1 M179. (**A**) Analysis of terpinen-4-ol. (**C**) Analysis of reaction mixture. (**B**) The MS spectra showed the base peak of terpinen-4-ol is 137.09 m/z. (**D**) The MS spectra showed the base peak of M1 is 153.07 m/z. (**E**) The MS spectra showed the base peak of M2 is 153.07 m/z.

Terpinen-4-ol and its metabolites were analyzed by LC-MS to characterize metabolites produced by CYP102A1 mutants. The LC-MS chromatogram and fragmentation are shown in Fig. 4. The base peak of terpinen-4-ol is m/z 137.09 (Fig. 4B). Analysis of metabolites of terpinen-4-ol exhibited two products of M1 and M2 with same m/z, 153.07 (Fig. 4D and 4E). These results indicate a 16-dalton difference in the molecular weight between terpinen-4-ol (m/z 137.09) and metabolites (M1 and M2; m/z 153.07). To sum up, M1 and M2 are assumed to be mono-hydroxylation products of terpinen-4-ol.

### Characterization of Metabolites of Terpinen-4-ol by GC-MS

A reaction mixture was analyzed by GC-MS to characterize terpinen-4-ol and its products by CYP102A1 mutants (Figs. S3 and S4). GC-MS analysis showed four peaks, M1–M4 (Fig. S3B). The fragmentation patterns (M1–M4) are shown in Fig. S4. We found that the fragmentation patterns of M3 and M4 were similar to those of terpinen-4-ol metabolites produced by human P450 [51, 52]—possibly not exactly the same, but it may be a clue for characterizing chemical structures of M3 and M4. To clarify which peaks were M1 and M2 in the GC chromatogram, M1 and M2 was separated by HPLC; C and D in Fig. S3 are GC traces of separated M1 and M2, respectively, proving the retention time of M1 and M2.

### Characterization of Metabolites of Terpinen-4-ol by NMR

Two major metabolites M1 and M2 were purified by HPLC. To ensure that M1 and M2 were purified, further HPLC analysis was conducted. The purities of M1 and M2 was confirmed as 99.8% and 99.9%, respectively, which were based on the area percentage (Fig. S5).

**Fig. 5:**
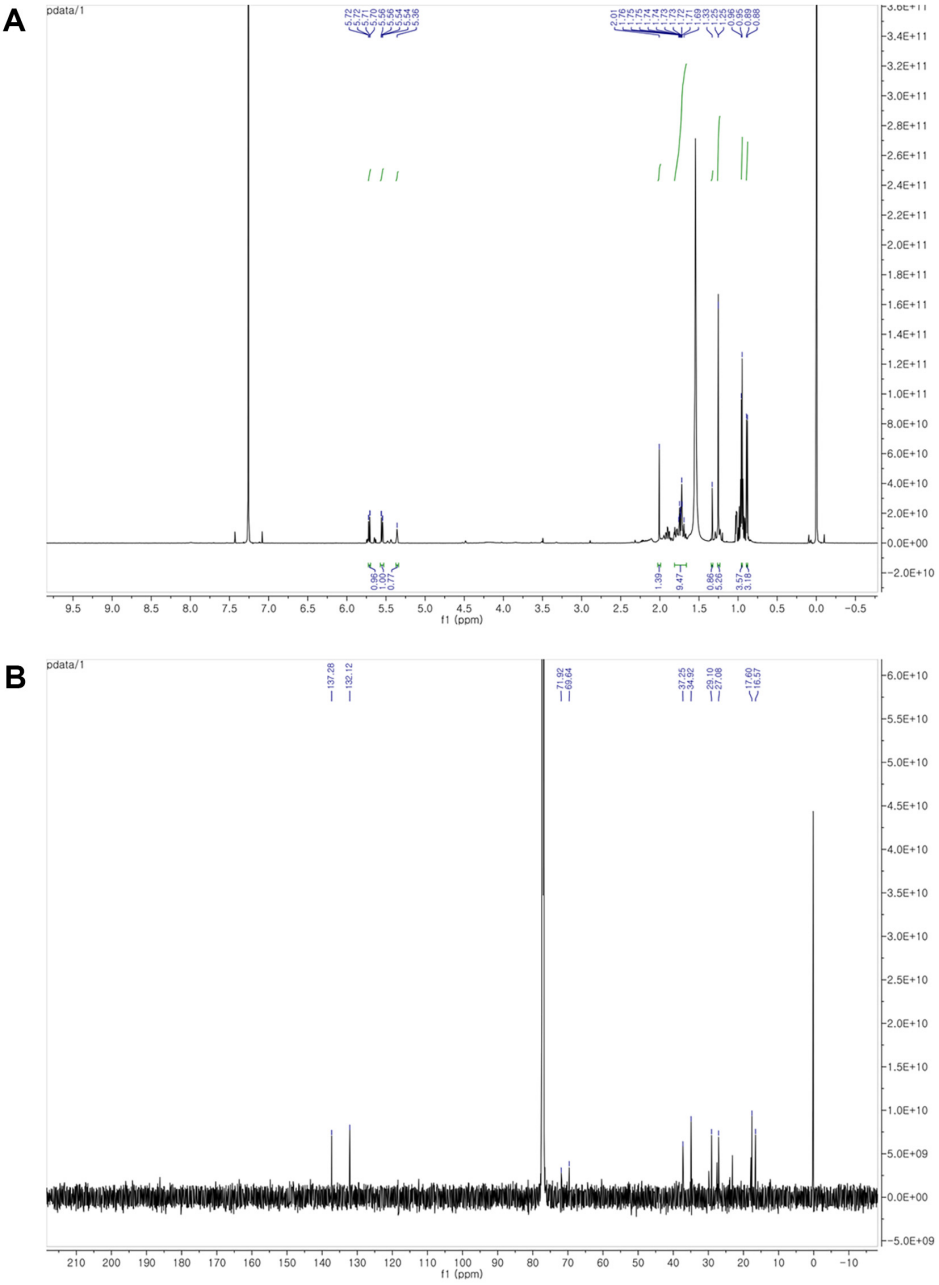
1D NMR spectrum of M1. (**A**) ^1^H NMR spectrum of M1 in CDCl_3_ (600 MHz). (**B**) ^13^C NMR spectrum of M1 in CDCl_3_ (150 MHz).

M1 was isolated as a colorless oil. ^1^H and ^13^C NMR analyses of M1 are summarized in Table S2. The ^1^H NMR spectrum of M1 in CDCl_3_ showed one pair olefinic proton signals at δ_H_ 5.71 (1H, dd, *J* = 9.6, 1.8 Hz, H-3) and 5.55 (1H, dd, *J* = 9.6, 1.8 Hz, H-2), two methylene protons at δ_H_ 1.60–1.68 (4H, m, H-5, 6), one methine at δ_H_ 1.54 (1H, m, H-8), and three methyl groups at δ_H_ 1.28 (3H, s, H-7), 0.95 (3H, d, *J* = 6.0 Hz, H-9), and 0.89 (3H, d, *J* = 6.0 Hz, H-8), which suggested the presence of the monoterpene backbone. In addition, the ^13^C NMR of M1 showed two quaternary carbons at δ_C_ 71.8 (C-4) and 69.6 (C-1). Consistent with these ^1^H NMR interpretations, ^13^C NMR and HSQC analyses of M1 further suggested that M1 comprised 1,4-dihydroxy-*p*-menth-2-ene three methyl-substituted *p*-menth-diol. The linkage points of the methyl residues in M1 were established unambiguously through the key HMBC correlations. The relative configurations of the pseudo-axial methyl on C-1 and the pseudo-equatorial isopropyl moiety on C-4 locations in the M1 were characterized by the NOESY spectrum. The spectrum of M1 showed relationships between H-7, H-9, and H-10, indicating a 1R*,4S* configuration between methyl and isopropyl residues. Thus, M1 was identified as (1R*,4S*)-1,4-dihydroxy-*p*-menth-2-ene through comparison of the physicochemical and spectroscopic data with those of reference data (Figs. 5, S6, and S7) [61-63]. Although we tried to obtain the NMR analysis of M2 by obtaining samples in the same way as M1, we could not obtain an NMR result.

### Kinetic Parameters of Formation of Terpinen-4-ol Metabolites Catalyzed by CYP102A1 Mutants

Kinetic parameters were analyzed using CYP102A1 M179 with various concentrations of terpinen-4-ol (0.5–10 mM) and incubation time of 10 min. Kinetic parameters (*K*_m_ and *k*_cat_) of terpinen-4-ol metabolites are shown in Fig. S8 and Table 1. The activities of WT CYP102A1 and CYP102A1 M179 were not compared to those of terpinen-4-ol, because WT CYP102A1 had no apparent activity concerning terpinen-4-ol. *K*_m_ values of M1 and M2 exhibited 3.4 mM and 3.2 mM, respectively; *k*_cat_ values of M1 and M2 exhibited 58.7 min^–1^ and 281 min^–1^, respectively. M2 showed a 4.8-fold higher *k*_cat_ value compared to M1. The catalytic efficiency (*k*_cat_/*K*_m_) of terpinen-4-ol metabolite formation by CYP102A1 M179 was 17 min^–1^∙mM^–1^ (M1) and 88 min^–1^∙mM^–1^ (M2), respectively.

### Spectral Binding Titration

**Fig. 6:**
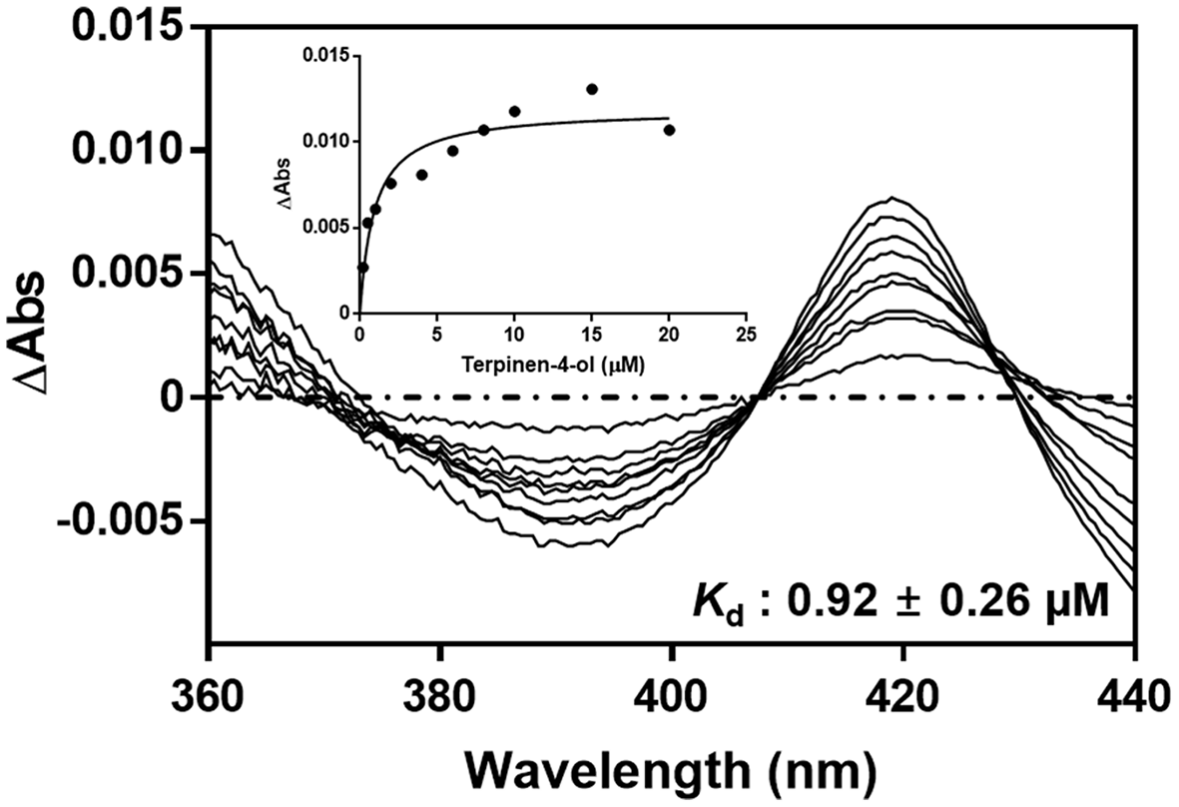
Spectral binding affinity of CYP102A1 M179 toward terpinen-4-ol. CYP102A1 M179 (2 μM) in 100 mM potassium phosphate buffer was titrated with various substrate concentrations. It was defined as a type II shift. The dissociation constant (*K*_d_) of terpinen-4-ol to CYP102A1 M179 was determined as 0.92 ± 0.26 μM.

Spectral binding titration was performed to exhibit terpinen-4-ol’s binding affinity with CYP102A1 M179. The binding of terpinen-4-ol to CYP102A1 M179 showed a decreased trend at 390 nm and an increasing trend at 420 nm. As a result, it was defined as a type II shift. The dissociation constant (*K*_d_) of terpinen-4-ol to CYP102A1 M179 was determined as 0.92 ± 0.26 μM (Fig. 6), showing that M179 has a high binding affinity to terpinen-4-ol when compared to *K*_m_ values.

### Time Course-Dependent Formation of Terpinen-4-ol Metabolites

**Fig. 7:**
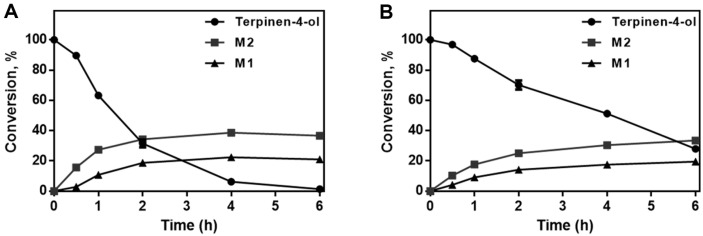
Time course-dependent formation of terpinen-4-ol metabolites. (**A**) The formation of terpinen-4-ol metabolites under 5 mM of terpinen-4-ol. (**B**) The formation of terpinen-4-ol metabolites under 5 mM of terpinen-4-ol. The reaction mixture of (**A**) and (**B**) contained 0.2 μM of CYP102A1 M179 in 100 mM potassium phosphate buffer (pH 7).

Incubation times from 30 min to 6 h were tested to find the best reaction time for yield and productivity. Formation of M1 and M2 by CYP102A1 M179 was conducted under various substrate conditions (5 mM and 10 mM). When the reaction mixture contained 5 mM, the formation of M1 and M2 was saturated at 4 h, and yields were 22.3% and 38.6%, respectively. When using 10 mM of substrate, formation of M1 and M2 increased slightly up to 6 h. At 6 h, the yields of M1 and M2 were 19.5% and 33.5% (Fig. 7).

In addition, as a control experiment the stability of terpinen-4-ol during reaction was tested. The mixture containing 5 mM of terpinen-4-ol in 100 mM potassium phosphate buffer (pH 7.4) was incubated from 30 min to 6 h. Terpinen-4-ol during incubation was slightly diminished depending incubation time (Fig. S9). The remaining concentrations of terpinen-4-ol at 1 h and 6 h were 91% and 63% when compared to the initial concentrations. At present the exact reason why the substrate decreased during incubation is not clear. Terpinen-4-ol itself is known to be volatile, but it is slightly soluble in water (387 mg/l = 2.51 mM) (https://www.thegoodscentscompany.com/data/rw1021751.html). Therefore, terpinen-4-ol does not seem to be volatile in water/DMSO (v/v, 98/2). Incubation for more than 1 h may lead to instability of terpinene-4-ol in aqueous solutions.

## Conclusion

In conclusion, we found that even though WT CYP102A1 had no apparent activity concerning terpinen-4-ol, engineered CYP102A1 displayed catalytic activity with terpinen-4-ol. Among tested mutants, M179 (R47L/F81I/F87V/E143G/L188Q/N213S/E267V) showed the highest activity concerning terpinen-4-ol. Engineered CYP102A1 could produce two major metabolites (M1 and M2). The characterization of metabolites formed by CYP102A1 mutants was confirmed using HPLC, LC-MS, GC-MS, and NMR. M1 was defined as 1,4-dihydroxy-*p*-menth-2-ene. Using LC-MS, we found that the fragmentation pattern of M2 showed a 16-dalton increase in molecular weight compared with terpinen-4-ol. Additionally, the peaks of M2 (135.08, 153.07, 154.07, and 194.07 m/z) were as those of M1 when comparing fragmentation patterns. In addition, the fragmentation pattern of GC-MS was similar between M1 and M2. These findings propose that the structures of two metabolites are likely to be similar. Therefore, M2 seems to be a mono-hydroxylated product. We also found that fragmentation patterns of M3 and M4 (Fig. S4) were similar to those of previous studies [51, 52]. Taken together, we propose the formation mechanism of 1,4-dihydroxy-*p*-menth-2-ene from terpinen-4-ol via epoxide and diol intermediates (Fig. S9) as described previously [49, 64]. We suggest that the mono-hydroxylated product is produced through intermediate epoxidation and that engineered CYP102A1 can be applied to biotechnology industries as a biocatalyst for the production of terpene derivatives.

## Supplemental Materials

Supplementary data for this paper are available on-line only at http://jmb.or.kr.



## Figures and Tables

**Table 1:** Kinetic parameters of hydroxylated metabolite formation from terpinen-4-ol using CYP102A1 M179.

Product	*k*_cat_ (min^-1^)	*K*_m_ (mM)	*k*_cat_/*K*_m_ (min^-1^•mM^-1^)
M1	58.7 ± 2.2	3.4 ± 0.3	17 ± 2.0
M2	281 ± 11	3.2 ± 0.3	88 ± 9.0

## References

[1] Carson CF, Hammer KA, Riley TV (2006). *Melaleuca alternifolia* (Tea Tree) oil: a review of antimicrobial and other medicinal properties. Clin. Microbiol. Rev..

[2] Homer LE, Leach DN, Lea D, Slade Lee L null, Henry RJ, Baverstock PR (2000). Natural variation in the essential oil content of *Melaleuca alternifolia* Cheel (Myrtaceae). Biochem. Syst. Ecol..

[3] Schnitzler P, Schön K, Reichling J (2001). Antiviral activity of Australian tea tree oil and eucalyptus oil against herpes simplex virus in cell culture. Pharmazie.

[4] Carson CF, Ashton L, Dry L, Smith DW, Riley TV (2001). *Melaleuca alternifolia* (tea tree) oil gel (6%) for the treatment of recurrent herpes labialis. J. Antimicrob. Chemother..

[5] Carson CF, Riley TV, Cookson BD (1998). Efficacy and safety of tea tree oil as a topical antimicrobial agent. J. Hosp. Infect..

[6] Williams LR, Asre S, Home VN (1994). Topical applications containing tea tree oil for vaginal conditions. Cosmet. Aerosols Toiletries Aust..

[7] Griffin SG, Wyllie SG, Markham JL, Leach DN (1999). The role of structure and molecular properties of terpenoids in determining their antimicrobial activity. Flavour Fragr. J..

[8] Zhang Y, Feng R, Li L, Zhou X, Li Z, Jia R (2018). The Antibacterial Mechanism of Terpinen-4-ol Against *Streptococcus agalactiae*. Curr. Microbiol..

[9] Hammer KA, Carson CF, Riley TV (1998). In-vitro activity of essential oils, in particular *Melaleuca alternifolia* (tea tree) oil and tea tree oil products, against *Candida* spp. J. Antimicrob. Chemother..

[10] Hammer KA, Carson CF, Riley TV (2002). In vitro activity of *Melaleuca alternifolia* (tea tree) oil against dermatophytes and other filamentous fungi. J. Antimicrob. Chemother..

[11] Kong Q, Qi J, An P, Deng R, Meng J, Ren X (2020). *Melaleuca alternifolia* oil can delay nutrient damage of grapes caused by *aspergillus ochraceus* through regulation of key genes and metabolites in metabolic pathways. Postharvest Biol. Technol..

[12] Burdock GA (2009). Fenaroli's Handbook of Flavor Ingredients.

[13] Api AM, Belsito D, Botelho D, Browne D, Bruze M, Burton GA (2017). RIFM fragrance ingredient safety assessment, 4-Carvomenthenol, CAS Registry Number 562-74-3. Food Chem. Toxicol..

[14] Bhatia SP, McGinty D, Letizia CS, Api AM (2008). Fragrance material review on 4-carvomenthenol. Food Chem. Toxicol. 46 Suppl.

[15] Liao M, Shi S, Wu H, Yang Q, Zhu Z, Xiao J (2020). Effects of terpinen-4-ol fumigation on protein levels of detoxification enzymes in *Tribolium confusum*. Arch. Insect Biochem. Physiol..

[16] Campolo O, Patanè V, Verdone AM, Palmeri V (2012). Survey of solid impurities and active infestation in flours produced in Calabria (Italy). J. Stored Prod. Res..

[17] Campolo O, Verdone M, Laudani F, Malacrinò A, Chiera E, Palmeri V (2013). Response of four stored products insects to a structural heat treatment in a flour mill. J. Stored Prod. Res..

[18] Cook DJ, Finnigan JD, Cook K, Black GW, Charnock SJ (2016). Cytochromes P450: history, classes, catalytic mechanism, and industrial application. Adv. Protein Chem. Struct. Biol..

[19] Omura T, Sato R (1962). A new cytochrome in liver microsomes. J. Biol. Chem..

[20] Omura T, Sato R (1964). THE CARBON MONOXIDE-BINDING PIGMENT OF LIVER MICROSOMES. I. EVIDENCE FOR ITS HEMOPROTEIN NATURE. J. Biol. Chem..

[21] Nam W, McCleverty JA, Meyer TJ (2003). 8.12 - Cytochrome P450. Comprehensive Coordination Chemistry II.

[22] Shankar K, Mehendale HM, Wexler P (2014). Cytochrome P450. Encyclopedia of Toxicology.

[23] Lynch T, Price A (2007). The effect of cytochrome P450 metabolism on drug response, interactions, and adverse effects. Am. Fam..

[24] Whitehouse CJC, Bell SG, Wong L-L (2012). P450(BM3) (CYP102A1): connecting the dots. Chem. Soc. Rev..

[25] Thistlethwaite S, Jeffreys LN, Girvan HM, McLean KJ, Munro AW (2021). A Promiscuous bacterial P450: the unparalleled diversity of BM3 in pharmaceutical metabolism. Int. J. Mol. Sci..

[26] Kokorin A, Parshin PD, Bakkes PJ, Pometun AA, Tishkov VI, Urlacher VB (2021). Genetic fusion of P450 BM3 and formate dehydrogenase towards self-sufficient biocatalysts with enhanced activity. Sci. Rep..

[27] Peters MW, Meinhold P, Glieder A, Arnold FH (2003). Regio- and enantioselective alkane hydroxylation with engineered cytochromes P450 BM-3. J. Am. Chem. Soc..

[28] Holec C, Hartrampf U, Neufeld K, Pietruszka J (2017). P450 BM3-catalyzed regio- and stereoselective hydroxylation aiming at the synthesis of phthalides and isocoumarins. Chembiochem.

[29] Hui C, Singh W, Quinn D, Li C, Moody TS, Huang M (2020). Regio- and stereoselectivity in the CYP450BM3-catalyzed hydroxylation of complex terpenoids: a QM/MM study. Phys. Chem. Chem. Phys..

[30] Huang X, Sun Y, Osawa Y, Chen YE, Zhang H (2023). Computational redesign of cytochrome P450 CYP102A1 for highly stereoselective omeprazole hydroxylation by UniDesign. J. Biol. Chem..

[31] Nguyen THH, Woo SM, Nguyen NA, Cha GS, Yeom SJ, Kang HS (2020). Regioselective hydroxylation of naringin dihydrochalcone to produce neoeriocitrin dihydrochalcone by CYP102A1 (BM3) mutants. Catalysts.

[32] Janocha S, Schmitz D, Bernhardt R (2015). Terpene hydroxylation with microbial cytochrome P450 monooxygenases. Adv. Biochem. Engin./Biotechnol..

[33] Urlacher VB, Girhard M (2012). Cytochrome P450 monooxygenases: an update on perspectives for synthetic application. Trends Biotechnol..

[34] Seifert A, Vomund S, Grohmann K, Kriening S, Urlacher VB, Laschat S (2009). Rational design of a minimal and highly enriched CYP102A1 mutant library with improved regio-, stereo- and chemoselectivity. Chembiochem.

[35] Seifert A, Antonovici M, Hauer B, Pleiss J (2011). An efficient route to selective bio-oxidation catalysts: an iterative approach comprising modeling, diversification, and screening, based on CYP102A1. Chembiochem.

[36] Ikebe J, Suzuki M, Komori A, Kobayashi K, Kameda T (2021). Enzyme modification using mutation site prediction method for enhancing the regioselectivity of substrate reaction sites. Sci. Rep..

[37] Venkataraman H, Beer SBA de, Geerke DP, Vermeulen NPE, Commandeur JNM (2012). Regio- and stereoselective hydroxylation of optically active α-ionone enantiomers by engineered cytochrome P450 BM3 mutants. Adv. Synth. Catal..

[38] Urlacher VB, Makhsumkhanov A, Schmid RD (2006). Biotransformation of beta-ionone by engineered cytochrome P450 BM-3. Appl. Microbiol. Biotechnol..

[39] Maurer SC, Schulze H, Schmid RD, Urlacher V (2003). Immobilisation of P450 BM-3 and an NADP+ cofactor recycling system: towards a technical application of heme-containing monooxygenases in fine chemical synthesis. Adv. Synth. Catal..

[40] Appel D, Lutz-Wahl S, Fischer P, Schwaneberg U, Schmid RD (2001). A P450 BM-3 mutant hydroxylates alkanes, cycloalkanes, arenes and heteroarenes. J. Biotechnol..

[41] Liu X, Kong JQ (2017). Steroids hydroxylation catalyzed by the monooxygenase mutant 139-3 from *Bacillus megaterium* BM3. Acta Pharm. Sin. B..

[42] Venkataraman H, Te Poele EM, Rosłoniec KZ, Vermeulen N, Commandeur JNM, van der Geize R (2015). Biosynthesis of a steroid metabolite by an engineered *Rhodococcus erythropolis* strain expressing a mutant cytochrome P450 BM3 enzyme. Appl. Microbiol. Biotechnol..

[43] Rea V, Kolkman AJ, Vottero E, Stronks EJ, Ampt K a M, Honing M (2012). Active site substitution A82W improves the regioselectivity of steroid hydroxylation by cytochrome P450 BM3 mutants as rationalized by spin relaxation nuclear magnetic resonance studies. Biochemistry.

[44] Ren Y, Liu S, Jin G, Yang X, Zhou YJ (2020). Microbial production of limonene and its derivatives: achievements and perspectives. Biotechnol. Adv..

[45] Hernandez-Ortega A, Vinaixa M, Zebec Z, Takano E, Scrutton NS (2018). A toolbox for diverse oxyfunctionalisation of monoterpenes. Sci. Rep..

[46] Sowden RJ, Yasmin S, Rees NH, Bell SG, Wong LL (2005). Biotransformation of the sesquiterpene (+)-valencene by cytochrome P450cam and P450BM-3. Org. Biomol. Chem..

[47] García-Carnelli C, Rodríguez P, Heinzen H, Menéndez P (2014). Influence of culture conditions on the biotransformation of (+)-limonene by *Aspergillus niger*. Z. Naturforsch. C J. Biosci..

[48] Kaspera R, Krings U, Pescheck M, Sell D, Schrader J, Berger RG (2005). Regio- and stereoselective fungal oxyfunctionalisation of limonenes. Z. Naturforsch. C J. Biosci..

[49] Garnes-Portolés F, López-Cruz C, Sánchez-Quesada J, Espinós-Ferri E, Leyva-Pérez A (2022). Solid-catalyzed synthesis of isomers-free terpinen-4-ol. Mol. Catal..

[50] Retajczyk M, Wróblewska A (2017). The isomerization of limonene over the Ti-SBA-15 catalyst-The influence of reaction time, temperature, and catalyst content. Catalysts.

[51] Haigou R, Miyazawa M (2012). Metabolism of (+)-terpinen-4-ol by cytochrome P450 enzymes in human liver microsomes. J. Oleo Sci..

[52] Miyazawa M, Haigou R (2011). Determination of cytochrome P450 enzymes involved in the metabolism of (-)-terpinen-4-ol by human liver microsomes. Xenobiotica.

[53] Shapira S, Pleban S, Kazanov D, Tirosh P, Arber N (2016). Terpinen-4-ol: a novel and promising therapeutic agent for human gastrointestinal cancers. PLoS One.

[54] Wu CS, Chen YJ, Chen JJW, Shieh JJ, Huang CH, Lin PS (2012). Terpinen-4-ol induces apoptosis in human nonsmall cell lung cancer in vitro and in vivo. Evid. Based Complement. Altern. Med..

[55] Nakayama K, Murata S, Ito H, Iwasaki K, Villareal MO, Zheng YW (2017). Terpinen-4-ol inhibits colorectal cancer growth via reactive oxygen species. Oncol. Lett..

[56] Graebin CS, Madeira M de F, Yokoyama-Yasunaka JKU, Miguel DC, Uliana SRB, Benitez D (2010). Synthesis and in vitro activity of limonene derivatives against *Leishmania* and *Trypanosoma*. Eur. J. Med. Chem..

[57] van Vugt-Lussenburg BMA, Stjernschantz E, Lastdrager J, Oostenbrink C, Vermeulen NPE, Commandeur JNM (2007). Identification of critical residues in novel drug metabolizing mutants of cytochrome P450 BM3 using random mutagenesis. J. Med. Chem..

[58] Carmichael AB, Wong LL (2001). Protein engineering of *Bacillus megaterium* CYP102. The oxidation of polycyclic aromatic hydrocarbons. Eur. J. Biochem..

[59] Cao NT, Cha GS, Kim JH, Lee Y, Yun CH, Nguyen NA (2023). Production of an O-desmethylated product, a major human metabolite, of rabeprazole sulfide by bacterial P450 enzymes. Enzyme Microb. Technol..

[60] Jang HH, Ryu SH, Le TK, Doan TTM, Nguyen THH, Park KD (2017). Regioselective C-H hydroxylation of omeprazole sulfide by *Bacillus megaterium* CYP102A1 to produce a human metabolite. Biotechnol. Lett..

[61] Rudbäck J, Bergström MA, Börje A, Nilsson U, Karlberg AT (2012). α-Terpinene, an antioxidant in tea tree oil, autoxidizes rapidly to skin allergens on air exposure. Chem. Res. Toxicol..

[62] Valente P, Avery TD, Taylor DK, Tiekink ERT (2009). Synthesis and chemistry of 2,3-dioxabicyclo[2.2.2]octane-5,6-diols. J. Org. Chem..

[63] Ahmed AA (2000). Highly oxygenated monoterpenes from *Chenopodium ambrosioides*. J. Nat. Prod..

[64] Li J, Gu W, Wang Z, Zhou X, Chen Y (2023). Asymmetric bio-epoxidation of unactivated alkenes. Chembiochem.

