# Supplementary material for: Production of Mono-Hydroxylated Derivatives of Terpinen-4-ol by Bacterial CYP102A1 Enzymes
Source: J Microbiol Biotechnol. 2023 Dec 1;34(3):725–34. doi: 10.4014/jmb.2310.10018 (PMC11016761; doi:10.4014/jmb.2310.10018)
Supplement: Supplementary file 1 [file jmb-34-3-725-supple.pdf]

J. Microbiol. Biotechnol.
https://doi.org/10.4014/jmb.2310.10018

Supplementary Tables and Figures

Production of Mono-hydroxylated Derivatives of Terpinen-4-ol by Bacterial CYP102A1 Enzymes

Jeong-Hoon Kim[1+], Chan Mi Park[2+], Hae Chan Jeong[1], Gyeong Han Jeong[3], Gun Su Cha[4], Sungbeom Lee[3,*], Chul-Ho Yun[2,5*]

[+]These authors contributed equally to this work.

[1]*School of Biological Sciences and Biotechnology, Graduate School, Chonnam National University, 77 Yongbongro, Gwangju 61186, Republic of Korea*

[2]*School of Biological Sciences and Technology, Chonnam National University, 77 Yongbongro, Gwangju 61186, Republic of Korea*

[3]*Research Division for Biotechnology, Advanced Radiation Technology Institute (ARTI), Korea Atomic Energy Research Institute (KAERI), 29 geumgu-gil Jeongeup, Jeollabuk-do 56212, Republic of Korea*

[4]*Namhae Garlic Research Institute, 2465-8 Namhaedaero, Namhae, Gyeongsangnamdo 52430, Republic of Korea*

[5]*Institute of Synthetic Biology for Carbon Neutralization, Chonnam National University, 77 Yongbongro, Gwangju 61186, Republic of Korea*

**Corresponding authors*:

E-mail: sungbeom@kaeri.re.kr, Phone: +82-63-570-3335, Fax: +82-63-570-3390

E-mail: chyun@chonnam.ac.kr, Phone: +82-62-530-2194, Fax: +82-62-530-2199

Table S1. Changed amino acid residues of the engineered CYP102A1 enzymes used in this study.

Enzyme	Changed amino acid(s)	References or GenBank accession number
1	F87A	[1]
2	A264G	[1]
3	F87A/A264G	[1]
4	R47L/Y51F	[1]
5	R47L/Y51F/A264G	[1]
6	R47L/Y51F/F87A	[1]
10	R47L/F87V/L188Q	[2]
14	R47L/F87V/E143G/L188Q/E267V	OR450073
15	R47L/E64G/F87V/E143G/L188Q/E267V	OR450074
16	R47L/F81I/F87V/E143G/L188Q/E267V	OR450075
159	R47L/F81I/F87V/E143G/L188Q/L262F/E267V	[3]
179	R47L/F81I/F87V/E143G/L188Q/N213S/E267V	OR450080
221	F11Y/R47L/F81I/F87V/E143G/L188Q/E267V/H408R	OR450081
225	D23G/R47L/F81I/F87V/E143G/L188Q/E267V/E409D	[3]
306	R47L/F81I/F87V/M112T/E143G/L188Q/E267V/M417T	[3]
371	D23G/R47L/F81I/F87V/F107L/D136G/E143G/L188Q/E267V	[3]
387	F11L/R47L/F81I/F87V/Q110P/E143G/L188Q/R190Q/E267V	OR450085
413	R47L/F81I/F87V/Q128R/E143G/L188Q/E267V/L287S/K309R/S383C	[3]
416	R47L/S72C/F81I/F87V/S108G/E143G/F158L/L188Q/M212V/E267V/E344D	OR450087
524	F42L/R47L/F81I/F87V/E143G/L188Q/E267V	OR450088
601	R47L/F81I/F87V/E143G/L150F/L188Q/E267V	OR450089
620	R47L/F81I/F87V/Q109L/E143G/L188Q/D199V/E267V	OR450090
788	R47L/F81I/F87V/K113N/E143G/L188Q/E267V/N319D/L347I/S383R	OR450093
850	F11Y/R47L/D68G/F81I/F87V/E143G/L188Q/E267V/H408R	OR450094

Table S2. ^{1}H and ^{13}C NMR shifts of M1[a]

Position	M1 ($CDCl_3$, 600 MHz)		Ref. ($CDCl_3$, 400 MHz)	
	δ_H (*J* in Hz)	δ_C, type	δ_H (*J* in Hz)	δ_C, type
1	–	69.6	–	67.2
2	5.55 (dd, 9.6, 1.8)	137.3	5.59 (dd, 10.0, 1.5)	135.4
3	5.71 (dd, 9.6, 1.8)	132.1	5.70 (dd, 10.0, 1.5)	133.4
4	–	71.9	–	71.5
5	1.60-1.68 (m)	27.1	1.88 (ddd, 14.0, 3.5)<br>1.52 (dddd, 14.0, 3.5, 3.0, 1.0)	27.0
6	1.60-1.68 (m)	34.9	1.82 (ddd, 14.0, 3.5)<br>1.68 (dddd, 14.0, 3.5, 3.0, 1.0)	33.4
7	1.28 (s)	29.1	1.32 (s)	29.5
8	1.54 (m)	37.3	1.70 (q, 7.0)	37.3
9	0.95 (d, 6.0)	16.5	0.94 (d, 7.0)	16.3
10	0.89 (d, 6.0)	17.6	0.86 (d, 7.0)	17.5

[a]NMR data of separated M1 was compared with references [4–6]

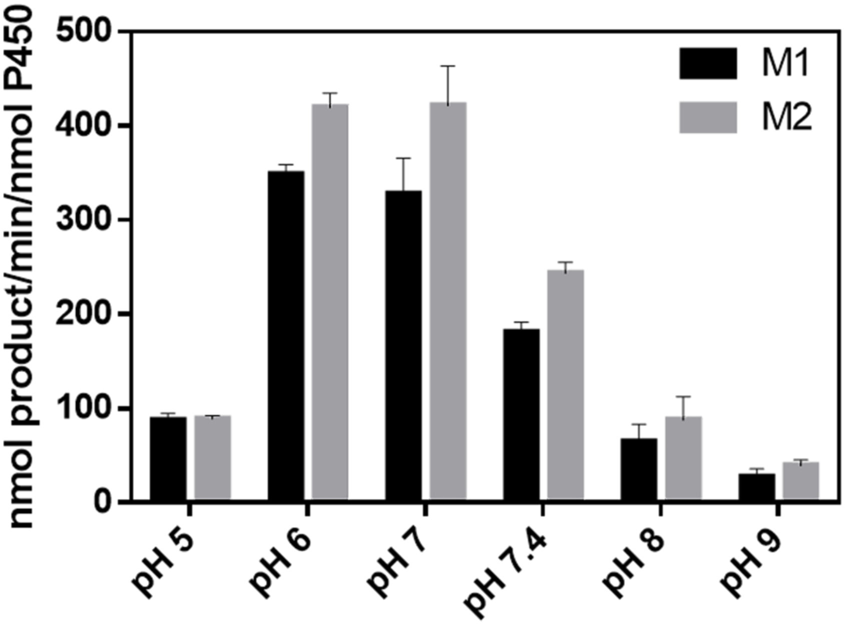

Fig. S1. Optimization of pH condition for production of terpinen-4-ol metabolites by CYP102A1 M179. The effect of pH for the metabolite formation by CYP102A1 M179 was investigated. The mixture contained 0.2 μM of CYP102A1 M179 and 5 mM of terpinen-4-ol in the range of pH 5-9 with 100 mM potassium phosphate buffer. The reaction mixture with NGS was incubated for 10 min for 35°C.

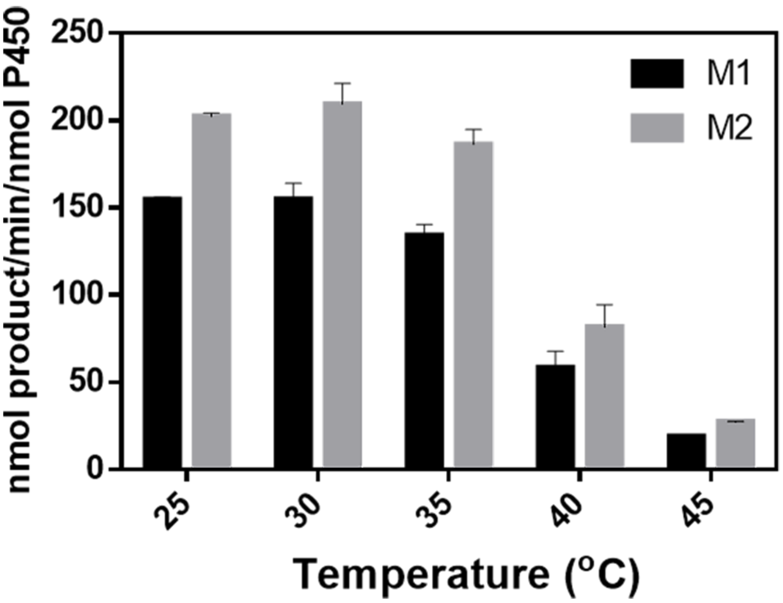

Fig. S2. **Optimization of temperature condition for production of terpinen-4-ol metabolites by CYP102A1 M179.** The effect of temperature for the metabolite formation by CYP102A1 M179 was investigated. The mixture contained 0.2 μM of CYP102A1 M179 and 5 mM of terpinen-4-ol in 100 mM potassium phosphate buffer (pH 7). The mixture with NGS was incubated for 30 min at indicated temperature.

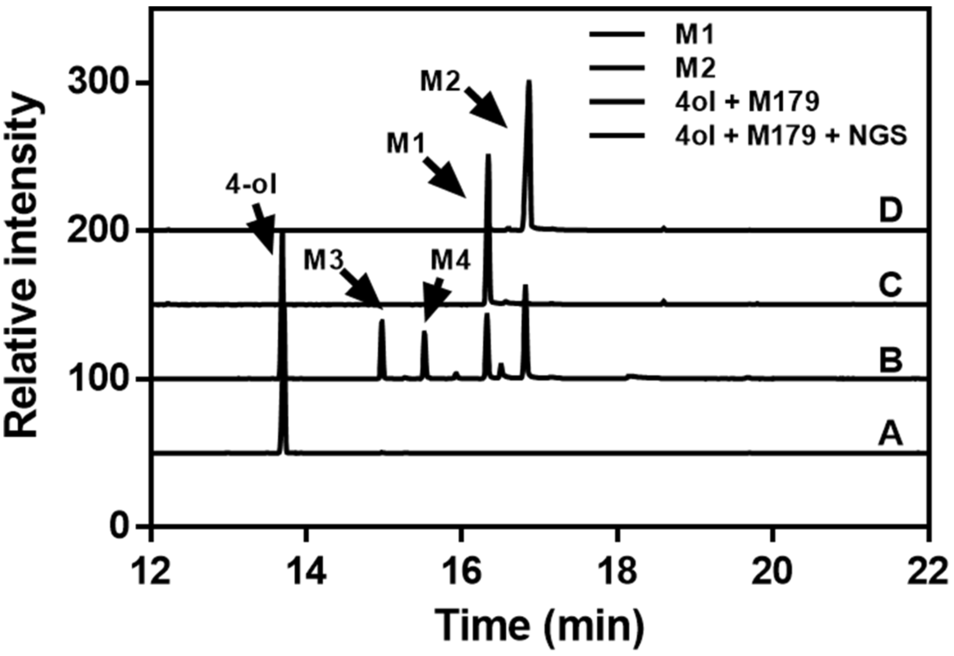

Fig. S3. GC chromatogram of terpinen-4-ol and its metabolites. (A) Terpinen-4-ol mixed with CYP102A1 M179. **(B)** Analysis of reaction mixture contained terpinen-4-ol, CYP102A1 M179, and NGS. Four peaks appeared compared with **(A). (C)** and **(D)** were from separated M1 and M2, respectively.

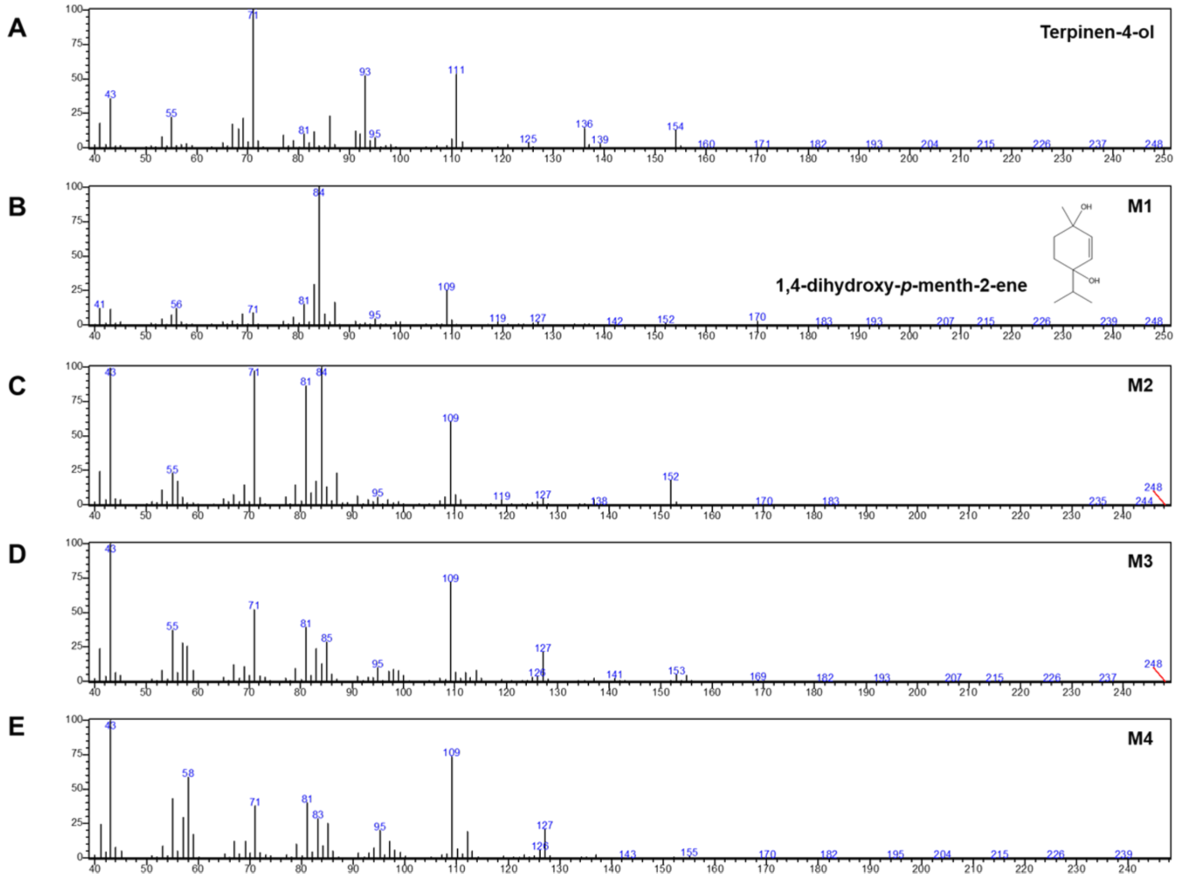

Fig. S4. **The fragmentation patterns of terpinen-4-ol and its metabolites using GC-MS.** These fragmentation patterns of M1-M4 are results of Figure S3B. M1 and M2 were the major metabolites in HPLC chromatogram. The fragmentation patterns of M3 and M4 were similar to the cases of the references [7,8].

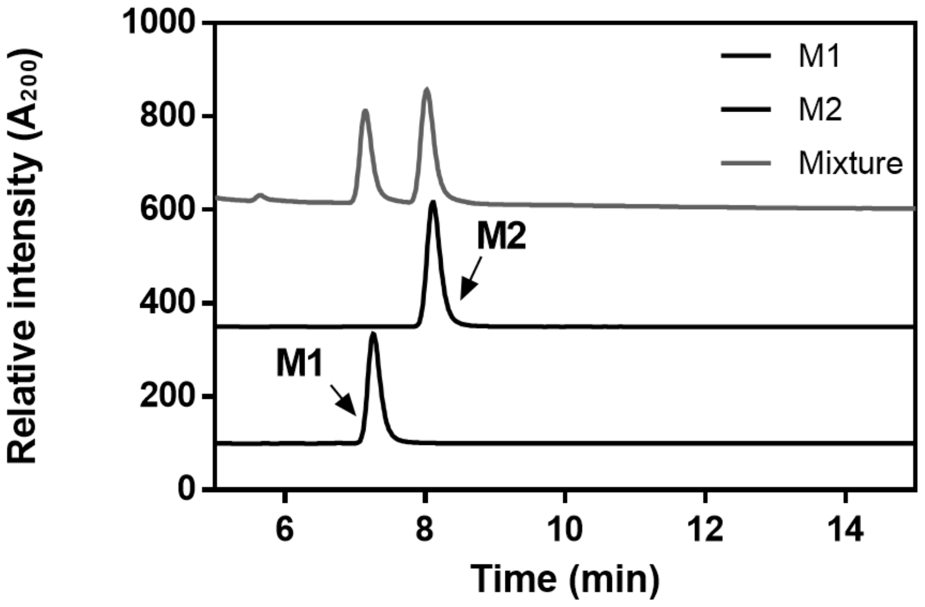

Fig. S5. **HPLC chromatogram of separated M1 and M2.** The purities of M1 and M2 was analyzed by HPLC. The purities of M1 and M2 were confirmed as 99.8% and 99.9%, respectively, which were calculated based on the area.

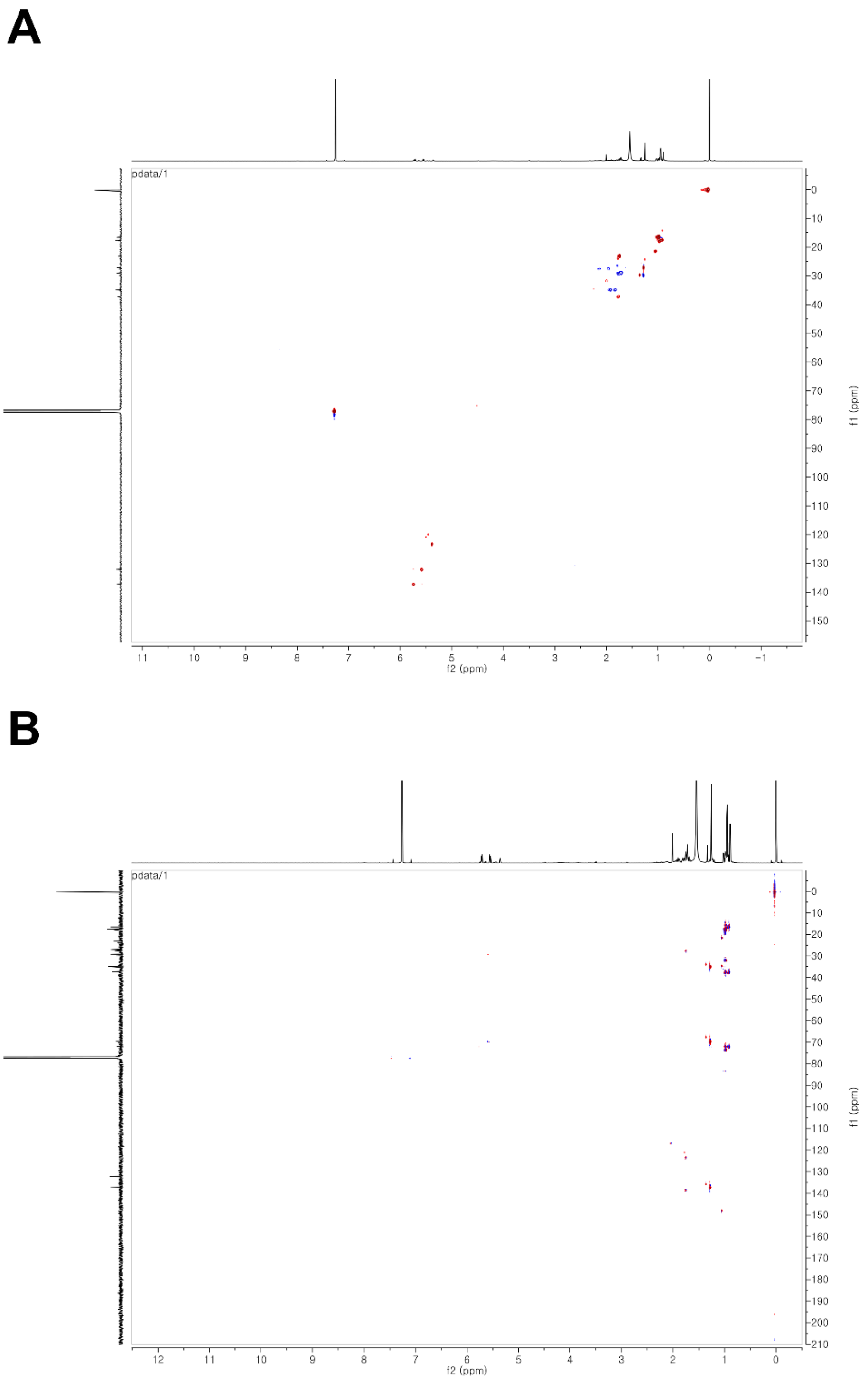

Fig. S6. **2D NMR spectrum of M1. (A)** HSQC spectrum of M1 in $CDCl_3$ (600 MHz). **(B)** HMBC spectrum of M-1 in $CDCl_3$ (600 MHz).

A

B

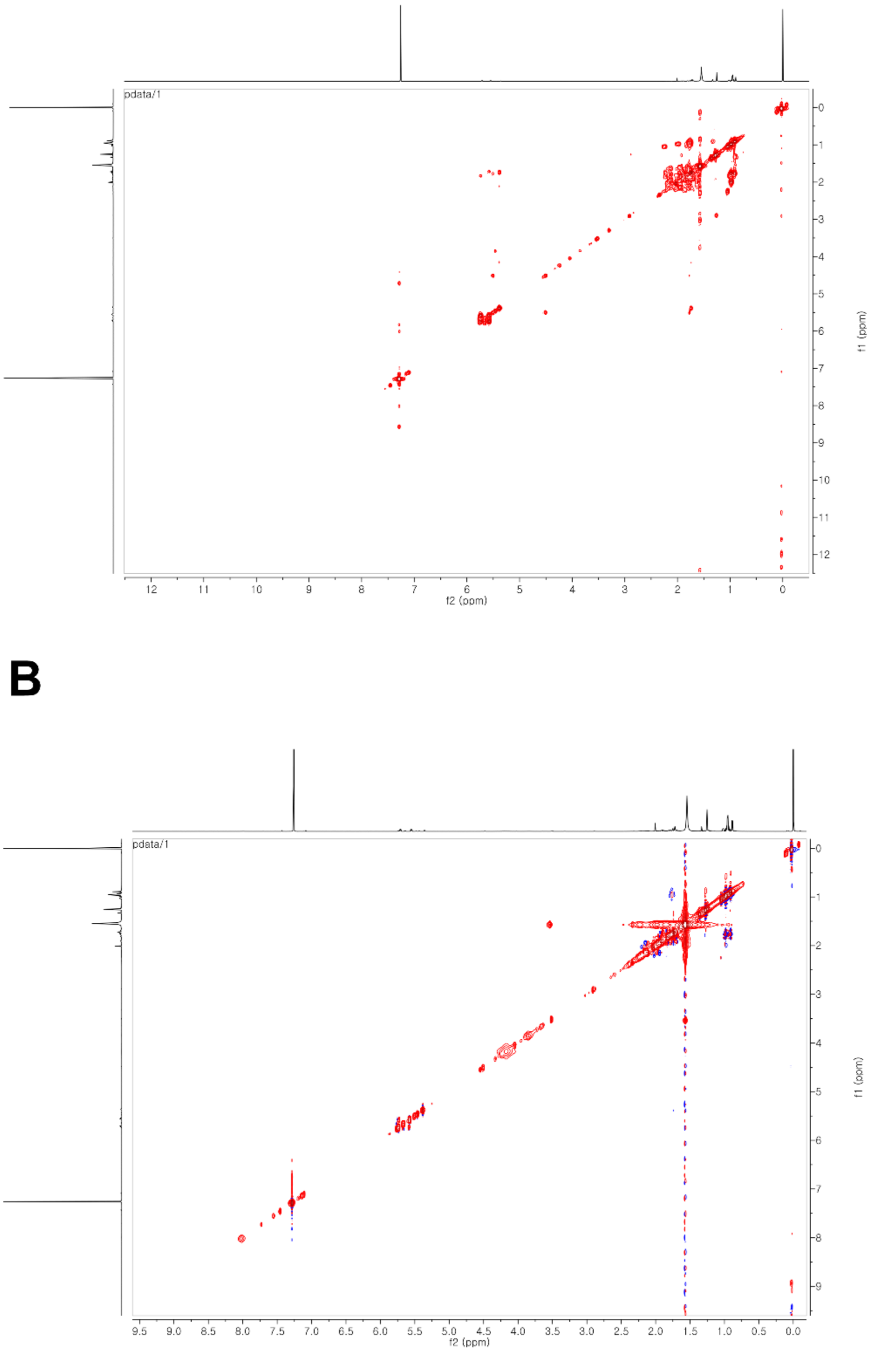

Fig. S7. **2D NMR spectrum of M1. (A)** ^{1}H-^{1}H COSY spectrum of M1 in $CDCl_3$ (600 MHz). **(B)** NOESY spectrum of M-1 in $CDCl_3$ (600 MHz).

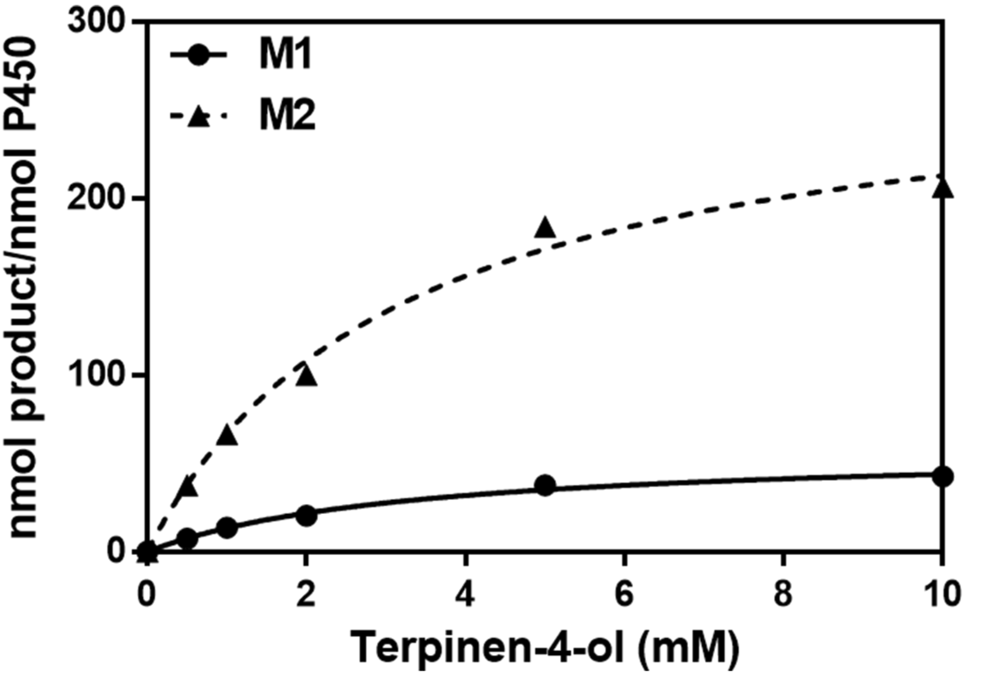

Fig. S8. **Kinetics of terpinen-4-ol metabolites formation from terpinen-4-ol by CYP102A1 M179.** The reaction mixture contained 0.2 μM of CYP102A1 M179 and various concentrations of terpinen-4-ol (0.5-10 mM) in 100 mM potassium phosphate buffer (pH 7). The reaction mixture with NGS was incubated for 10 min at 30°C.

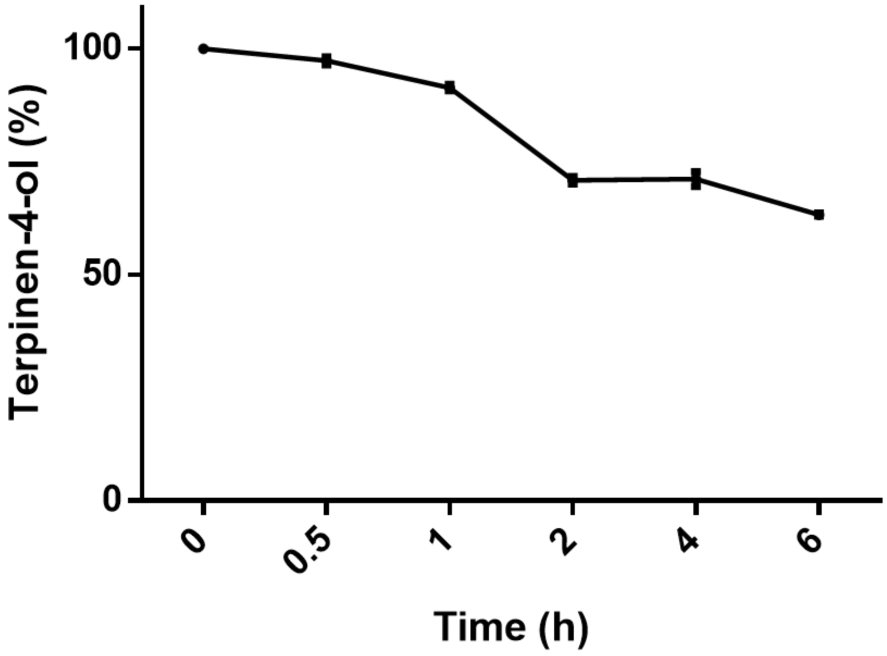

Fig. S9. Stability of terpinen-4-ol (5 mM) during incubation in 100 mM potassium phosphate (pH 7.4) at 30°C.

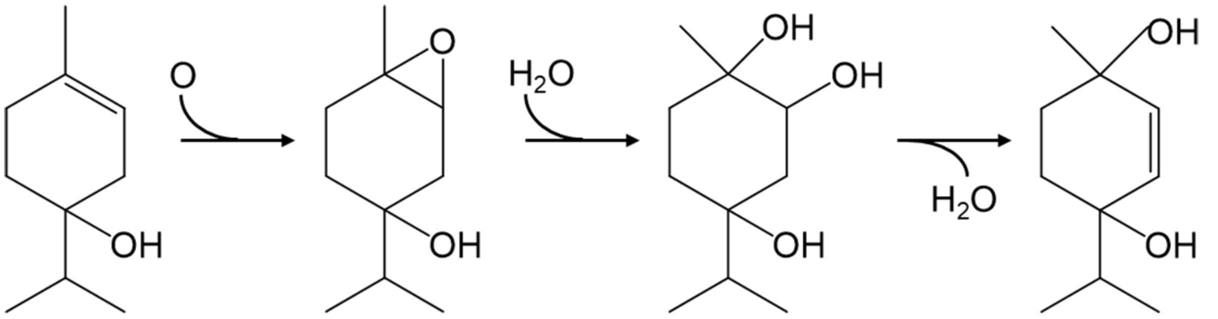

Fig. S10. **Proposed mechanism for the formation of 1,4-dihydroxy-*p*-menth-2-ene from terpinen-4-ol catalyzed by CYP102A1.**

References

1. Carmichael AB, Wong LL. 2001. Protein engineering of Bacillus megaterium CYP102. The oxidation of polycyclic aromatic hydrocarbons. *Eur. J. Biochem.* **268**: 3117–3125.

2. van Vugt-Lussenburg BMA, Stjernschantz E, Lastdrager J, Oostenbrink C, Vermeulen NPE, Commandeur JNM. 2007. Identification of critical residues in novel drug metabolizing mutants of cytochrome P450 BM3 using random mutagenesis. *J. Med. Chem.* **50**: 455–461.

3. Jang H-H, Ryu S-H, Le T-K, Doan TTM, Nguyen THH, Park KD, *et al.* 2017. Regioselective C-H hydroxylation of omeprazole sulfide by Bacillus megaterium CYP102A1 to produce a human metabolite. *Biotechnol. Lett.* **39**: 105–112.

4. Rudbäck J, Bergström MA, Börje A, Nilsson U, Karlberg A-T. 2012. α-Terpinene, an antioxidant in tea tree oil, autoxidizes rapidly to skin allergens on air exposure. *Chem. Res. Toxicol.* **25**: 713–721.

5. Valente P, Avery TD, Taylor DK, Tiekink ERT. 2009. Synthesis and chemistry of 2,3-dioxabicyclo[2.2.2]octane-5,6-diols. *J. Org. Chem.* **74**: 274–282.

6. Ahmed AA. 2000. Highly oxygenated monoterpenes from Chenopodium ambrosioides. *J. Nat. Prod.* **63**: 989–991.

7. Haigou R, Miyazawa M. 2012. Metabolism of (+)-terpinen-4-ol by cytochrome P450 enzymes in human liver microsomes. *J. Oleo Sci.* **61**: 35–43.

8. Miyazawa M, Haigou R. 2011. Determination of cytochrome P450 enzymes involved in the metabolism of (-)-terpinen-4-ol by human liver microsomes. *Xenobiotica* **41**: 1056–1062.
